# Yield Optimisation of Hepatitis B Virus Core Particles in *E. coli* Expression System for Drug Delivery Applications

**DOI:** 10.1038/srep43160

**Published:** 2017-03-03

**Authors:** Izzat Fahimuddin Bin Mohamed Suffian, Mitla Garcia-Maya, Paul Brown, Tam Bui, Yuya Nishimura, Amir Rafiq Bin Mohammad Johari Palermo, Chiaki Ogino, Akihiko Kondo, Khuloud T. Al-Jamal

**Affiliations:** 1 Institute of Pharmaceutical Science, King’s College London, Franklin-Wilkins Building, 150 Stamford Street, London SE1 9NH, UK; 2 Randall Division of Cell & Molecular Biophysics, King’s College London, New Hunt’s House, London SE1 1UL, UK; 3 Biomolecular Spectroscopy Centre, King’s College London, The Wolfson Wing, Hodgkin Building, SE1 1UL, UK; 4 Department of Chemical Science and Engineering, Graduate School of Engineering, Kobe University, 1-1 Rokkodai, Nada, Kobe 657-8501, Japan; 5 Barts and The London School of Medicine and Dentistry, Queen Mary University of London, 4 Newark Street, London E1 2AT, UK

## Abstract

An *E. coli* expression system offers a mean for rapid, high yield and economical production of Hepatitis B Virus core (HBc) particles. However, high-level production of HBc particles in bacteria is demanding and optimisation of HBc particle yield from *E. coli* is required to improve laboratory-scale productivity for further drug delivery applications. Production steps involve bacterial culture, protein isolation, denaturation, purification and finally protein assembly. In this study, we describe a modified *E. coli* based method for purifying HBc particles and compare the results with those obtained using a conventional purification method. HBc particle morphology was confirmed by Atomic Force Microscopy (AFM). Protein specificity and secondary structure were confirmed by Western Blot and Circular Dichroism (CD), respectively. The modified method produced ~3-fold higher yield and greater purity of wild type HBc particles than the conventional method. Our results demonstrated that the modified method produce a better yield and purity of HBc particles in an *E. coli*-expression system, which are fully characterised and suitable to be used for drug delivery applications.

The past quarter century has brought biomedical and health sciences researchers to focus on nanotechnologies. Cross-disciplinary research bringing together engineering, biology, physics and chemistry resulted in the production of a vast and diverse number of nanovectors, which have been explored in drug delivery and imaging^1^. In nanotechnology, 1–100 nm diameter particles are referred to as nanoparticles. Cancer-related nano-technology studies have shown substantial development in producing nanoparticles with different sizes, chemical nature and structure, all of which influence their applications including administration for targeted therapeutic and imaging moieties^2^,^3^. Nanoparticles can be developed from different materials: organic, inorganic or biological (macro) molecules. The latter, referred to as bio-nanoparticles can be formed by assembly of protein subunits^4^. One popular example is ferritin, a naturally occurring human protein, which is able to self-assemble into spherical capsids or cages^5^. However, the most promising protein cages are considered to be virus-derived nanoparticles or virus-like particles (VLPs). VLPs are self-assembling, non-replicative and non-pathogenic particles, which structurally mimic the viral capsid lacking the viral genome^6^. They can either be isolated directly after the coat protein expression in eukaryotic cells, or assembled *in vitro* from coat protein subunits purified from any recombinant host; ranging from mammalian cells to bacteria^7^,^8^.

As the first VLP candidate and the first icosahedral VLP carriers, HBc particles remain the most flexible and promising model for knowledge-based display of foreign peptide sequences^9^,^10^. HBc particles are hollow nanoparticles ranging from 30 to 34 nm in diameter with 7 nm thick envelopes^11^. These core particles are icosahedral nucleocapsids with primarily triangulation number T = 3 or T = 4 symmetry, each containing 180–240 units of 21 kDa core monomers^12^. Their self-assembly property and ability to assemble and dis-assemble freely through altering the urea concentration, modifying salt concentration and reducing disulfide bonds^13^,^14^,^15^, have been utilised to allow for encapsulation of various drugs^16^ and other biomolecules including nuclease^17^, oligonucleotides^18^ and siRNA^19^.

HBc particles can be expressed in homologous and heterologous expression systems. *Escherichia coli* offers a means for rapid, high yield and economical production of recombinant proteins^20^,^21^. However, high-level production of recombinant bio-nanoparticles in bacteria on a laboratory-scale is challenging. Challenges include: (1) biomass production of specific-gene selection cells^22^, (2) degradation of protein by host cell proteases^23^, (3) protein isolation from inclusion bodies^24^ and (4) successful protein folding^25^. Therefore, a yield optimisation study of HBc particles expression in *E. coli* is required to improve the productivity of the laboratory-scale for further drug delivery applications.

This study focused on developing an improved method (I) for producing wild type HBc (WT-HBc) particles in high yields, suitable for drug delivery applications. For comparison, WT-HBc particles were also prepared by the “conventional” method (C) reported previously by others^26^, Production steps involve bacterial culture, protein isolation, denaturation, purification and finally VLP assembly. The protein yield and purity were determined by Nanodrop™. HBc particle morphology was confirmed by Atomic Force Microscopy (AFM). Protein specificity and secondary structure were confirmed by Western Blot and Circular Dichroism (CD), respectively. We concluded that the modified method produced ~3 fold higher yield and greater purity of HBc particles than the conventional method.

## Results

### Optimisation of the WT-HBc particles expression and assembly method

**Figure 1:**
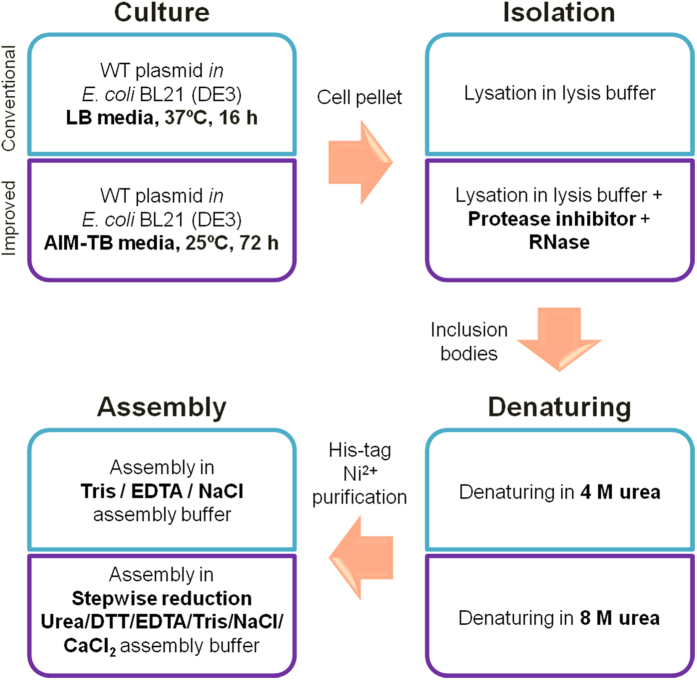
A scheme summarising WT-HBc particles production in *E. coli* expression system. WT-HBc particles were prepared using one of the two methods; the “conventional” or “improved” method.

In this study, we aimed at developing an improved method (I) for producing wild type HBc (WT-HBc) particles in high yields, suitable for drug delivery applications. For comparison, WT-HBc particles were also prepared by the “conventional” method (C) reported previously by others^26^. The four steps involved including bacterial culture, protein isolation, denaturing and assembly are summarised in Fig. 1. The major differences between the two methods and the rationale for these modifications will be highlighted here.

*E. coli* was chosen as the host organism to produce HBc particles on a laboratory scale, due to the advantages of relatively simple fermentation design and high final cell densities^20^. Auto-Induction Media Terrific Broth (AIM-TB) was used in the new method to replace Luria-Bertani Broth (LB) used previously. AIM-TB supports *E. coli* growth to a cell density 10-fold or higher in a shake flask^26^,^27^.

A protease inhibitor and RNase were included in the lysis buffer of the new method (Fig. 1), specifically to protect HBc proteins from cleavage by endogenous proteases and to remove contaminating RNA^28^,^29^.

Urea is used as a denaturing agent to solubilise the inclusion bodies containing WT-HBc particles. In the “conventional” method, a moderate urea concentration (4 M) in dissociation buffer was used compared to 8 M used in the new method. The higher urea concentration was expected to denature HBc particles to monomers more efficiently^13^. The His-tag, located at the C-terminus of HBc protein, resides inside the fully assembled particles^30^,^31^, so efficient denaturing of the HBc particles makes the His-tag more available for His-tag affinity chromatography, used in a later step of purification.

Purified WT-HBc protein, in the monomer form, was then re-folded *via* the assembly step to restore the core particles structure. In the “conventional” method, after affinity chromatography urea was removed by dialysis against Tris/EDTA/NaCl assembly buffer, where pronounced precipitation was associated with the sudden drop in urea concentration. In the new method; the purified WT-HBc monomers were re-folded by a stepwise reduction in urea concentration first to 2 M during column elution then to 0.5 M in dialysis against assembly buffer (DTT/EDTA/Tris/NaCl/CaCl_2_) then dialysis against buffer containing no urea. This altered assembly strategy was designed to improve the efficiency of protein re-assembly by reducing random protein folding, induced by disulfide bonds formation^32^,^33^, thereby increasing the yield of appropriately assembled WT-HBc particles.

### Protein yield, particle morphology and size

**Figure 2:**
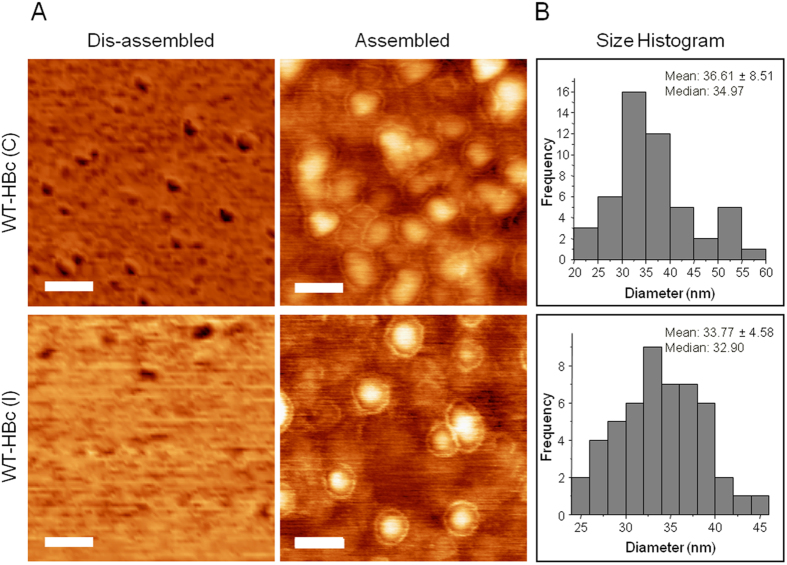
Morphological analysis of purified HBc core particles with Atomic Force Microscopy (AFM). (**A**) AFM images using tapping mode AFM (TM-AFM) and (**B**) Histogram analysis of assembled HBc particles. HBc particles were deposited on the mica substrates and measurements were carried out in air at 25 °C, using a Bruker Dimension ICON with Scan Assist. Dis-assembled HBc were achieved by dilution in distilled water at 40 °C for 10 min. Core shell structure was observed for the assembled particles for both formulations. Histograms were obtained for n = 50, analysed using WSxM v5.0 Developed 6.2 and Origin 7.5 software. Scan size is 300 nm × 300 nm. Scale bars are 60 nm.

**Figure 3:**
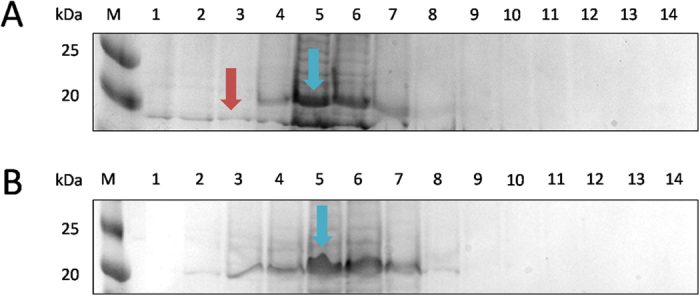
Elution profile of WT-HBc monomers from Ni^2+^-chelate affinity chromatography column (cropped gels). A column with 6 ml of cOmplete™ His-Tag Purification Resin was equilibrated with 3-times bed-volume (18 ml) of the dissociation buffer. The column was loaded with the protein probe and washed with 18 ml dissociation buffer. Bound proteins were eluted with 14 ml of elution buffer and collected in 1 ml fractions. The aliquots of each fractions were subjected to SDS-PAGE gel electrophoresis and stained with Coomassie Brilliant Blue. M, marker in kDa; Numbers 1–14 represent aliquots of the respective elution fractions for **(A)** WT-HBc (C) or **(B)** WT-HBc (I) monomers. Blue arrows represent WT-HBc monomers (21 kDa). Higher number of fractions (7 fractions, lane 2–8) were collected in case of WT-HBc (I) compared to WT-HBc (C) (5 fractions, lane 4–8). Some unknown protein bands were observed at molecular weight lower than 20 kDa (red arrow).

Significantly higher protein yields (p < 0.001) from 1 l growth medium, were obtained for WT-HBc prepared using the new method (3.21 ± 0.21 mg) compared with the conventional method (1.21 ± 0.30 mg) (Table 1). Height AFM images of HBc prepared by both methods revealed relatively monodisperse and spherical structures, observed only in case of the assembled particles. The “halo” observed suggested the presence of core-shell type structures. HBc mean diameters measured by AFM were 36.61 ± 8.51 nm and 33.77 ± 4.58 nm (p = 0.45 (>0.05)), respectively (Fig. 2). The OD_260_/OD_280_ values, used to determine the purity of the protein from nucleic acids contamination, are shown in Table 1. WT-HBc (I) showed a significantly lower value of OD_260_/OD_280_ ratio (0.58 ± 0.01) than WT-HBc (C) (1.02 ± 0.04) suggesting the higher nucleic acid contamination in the latter. A OD_260_/OD_280_ ratio greater than 0.6 indicates nucleic acid contamination in the protein samples^34^. In addition, as shown in Fig. 3 (cropped gels) and Supplementary Information Figure S1 (full length gels), some unknown protein bands were observed on SDS-PAGE at molecular weight lower than 20 kDa, in case of WT-HBc (C) (Fig. 3A, Supplementary Figure S1a), but not for WT-HBc (I) (Fig. 3B, Supplementary Figure S1b). This result confirmed the impurities observed in the WT-HBc (C) samples. Overall, these results indicated that the new method provides HBc particles of an improved yield and purity.

### Protein specificity and secondary structure analysis of the purified WT-HBc particles

**Figure 4:**
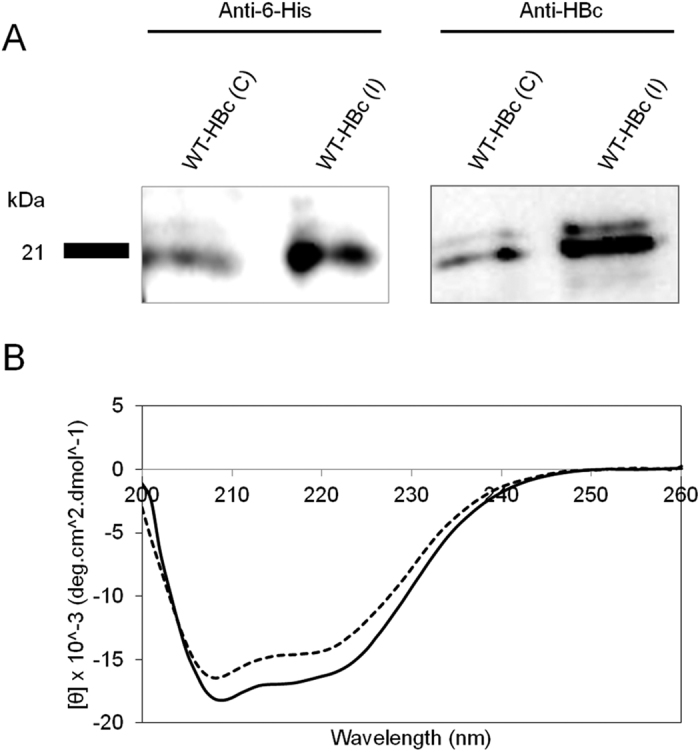
Protein specificity and secondary structure analysis of HBc particles. (**A**) Western blotting (cropped blot) and **(B)** Circular Dichroism (CD) analysis of WT-HBc particles. Denatured HBc samples were subjected to SDS-PAGE followed by immuno-blotting using anti-6-His and anti-HBc antibodies. Results confirmed the presence of specific protein bands at 21 kDa. CD graph shows the overall conformation of far-UV CD analysis of WT-HBc core particles. WT-HBc (C) (solid line) and WT-HBc (I) (dashed line) exhibited spectra typical of α-helix-containing proteins with minima at 220 and 208 nm. The CD spectra are typical of WT-HBc core monomer secondary structure.

Western blot analysis was performed to confirm the protein specificity of WT-HBc particles. The presence of HBc antigen and His-tag, as biochemical markers, was confirmed. Specific protein bands, positive for anti-6-His and anti-HBc antibodies, were observed at 21 kDa^26^ (Fig. 4A (cropped blot), Supplementary Information Figure S2 (full length blots)) confirming the successful expression of WT-HBc particles by both methods.

The typical secondary structure of the WT-HBc^35^ monomer was confirmed by far-UV circular dichroism (CD). WT-HBc particles from both preparations displayed a predominantly α-helix conformation with well-defined characteristic negative bands at 208 and 222 nm^35^ (Fig. 4B).

## Discussion

HBc particles have been expressed extensively in several types of hosts including *E. coli*^36^. However, it has been a challenge to produce a high yield of purified HBc particles on a laboratory-scale. In the conventional method of HBc particles production in *E. coli,* LB media is used as a culture medium^37^. LB media can support *E. coli* growth at an OD_600_ 0.7–0.8 under normal shaking incubation conditions^38^. However due to its mid-exponential growth phase, the pH of the cultures drops to pH 4 to 5 upon saturation, causing the antibiotics such as ampicillin to be degraded, resulting in a partial or no selection of cells. Under these conditions, 80% of the total cell population may not contain the intended plasmid, preventing these cells from being induced to express the target protein, resulting in low protein yields^23^. One approach to overcome this problem is to use an auto-induction media such as the AIM-TB media^39^. Auto-induction is based on the function of *lac* operon regulatory elements using a mixture of glucose, glycerol and lactose under diauxic growth conditions. During the initial growth period, glucose is preferentially used as a carbon source as a first sugar source. As the glucose is depleted, usually in mid to late log phase, the second source of sugar, lactose is taken up and converted by β-galactosidase to the inducer allolactose. Allolactose causes the release of *lac* repressor from its specific binding sites in the DNA, which induces the expressions of T7 RNA polymerase from the lacUV5 promoter, which thereby unblocks T7lac promoters, allowing expression of the target protein by T7 RNA polymerase. This transition from the un-induced to induced state under metabolic control of the expression host is one of the factors contributed to the higher amount of protein expressed in the bacteria. Furthermore, the increased richness of the AIM-TB media endorse to the higher amount of lysate harvested^23^.

Inclusions of protease inhibitors and RNase in a lysis buffer might be the important parameters for HBc isolation from the *E. coli* lysate. Bacterial cells contain many different types of proteases. As soon as cells are disrupted, proteases are released and can quickly degrade any protein, causing a drastic reduction in the protein yield during isolation and purification. Therefore, inclusion of protease inhibitors in the new method prevents their proteolytic activity which can cause cleavage of HBc protein fractions^28^. HBc particles can be contaminated with the host RNA when expressed in an *E. coli* expression system^40^. Addition of RNase in the early stage of purification removes contaminating RNA, hence preventing any remnant non sequitur activity for further studies^29^.

In this study, we isolated WT-HBc particles from protein inclusion bodies, which are insoluble protein aggregates, that can be easily separated by centrifugation from the rest of the soluble bacterial cytoplasmic proteins^41^. In order to solubilise the inclusion bodies, the extraction of protein is generally performed in the presence of denaturing agents. Here, a strategy of urea usage has been chosen for its compatibility with protein folding and most chromatographic procedures^42^,^43^. In the “conventional” method, a moderate urea concentration (4 M) in dissociation buffer was only able to solubilise the protein inclusion bodies partially with undissolved material observed after overnight incubation. However, in the new method, with 8 M urea in the dissociation buffer, the protein inclusion bodies were completely solubilised and dissociated WT-HBc proteins. A high concentration of urea acts to enhance the aqueous solubility of protein by weakening the hydrophobic interactions and stabilising the solvation of the unfolded protein. This allows a greater number of non-polar side chains to be exposed to water molecules, thus resulting in a completely solubilised protein in solution^44^,^45^.

The abrupt removal of urea to re-fold HBc monomers by dialysis against Tris/EDTA/NaCl assembly buffer led to precipitation, most probably due to the random proteins re-folding, which may have resulted in a low yield of the purified assembled WT-HBc particles. Here, a strategy of adding a reducing agent, DTT and a divalent salt, CaCl_2_ was used in the new method, helping to reduce the protein aggregation problem. DTT, a thiol reagent was used to reduce the disulfide bonds of proteins, which helps to prevent intra-molecular and intermolecular disulfide bonds from forming between cysteine residues of the protein. Ca^2+^ ion can also promote HBc particles assembly. It is suggested that the ionic strength from the calcium ions increases the electrostatic attractions between the proteins monomers thus act as an electrostatic switch for the protein self-assembly^46^. So when a highly concentrated WT-HBc monomers solution was dialysed under these conditions, the proteins re-folded at the correct geometry bonding, stabilising the protein structure, resulting in high re-folding yield of HBc particles and fewer aggregation problems^16^,^47^,^48^.

Another study by Zlotnick *et al*. demonstrated that the usage of assembly effectors could enhance HBc particles assembly. The usage of HAP1 [methyl 4-(2-chloro-4-fluorophenyl)-6-methyl-2-(pyridin-2-yl)- 1,4-dihydropyrimidine-5-carboxylate], a type of heteroaryldihydropyrimidine molecules, together with 150 mM NaCl was found to accelerate HBc particles assembly kinetics, resulting in 65% particles assembly, compared with particles assembled in 150 mM NaCl only (25% assembly)^49^,^50^. The addition of assembly effector in the future might be a good approach to improve protein assembly yield of HBc particles.

The organisation of HBc particles is known to be largely α-helical and quite different from previously known viral capsid proteins with β-sheet jellyroll packings^51^,^52^. Association of two amphipathic α-helical hairpins results in the formation of a dimer with a four-helix bundle as the major central feature. The four-helix bundles protrude, forming spikes, presents as a central part of major immudominant region of the HBc particles^53^. Here, both WT-HBc particles exhibited typical spectra of α-helix-containing proteins structure.

## Conclusion

The experimental work has focused on the initial development of functional nano-assemblies of virus-like particles for therapeutic applications in addition to the development of “proposed” novel biocompatible and therapeutically efficient nanocarriers for cancer. We demonstrated an improved method to produce a better yield and purity of HBc particles in an *E. coli*-expression system. The HBc particles produced are fully characterised and are suitable to be used to encapsulate drugs/biomolecules or to be bioengineered with targeting molecules.

## Materials

Luria-Bertani-Broth Miller, tryptone, yeast extract powder, MOPS, ampicillin sodium, IPTG dioxan free, AIM-terrific broth base including trace elements, Tris base Ultra-Pure, EDTA disodium and dithiothreitol (DTT) were obtained from ForMedium™ (UK). Urea (carbamide) were obtained from Melford (UK). Imidazole, Glycerol, 2-mercapoethanol, *N, N, N’, N’*-tetramethylethylenediamine, RNase A (from bovine pancreas) and bromophenol blue were obtained from Sigma Life Science (UK). Complete™ ULTRA Tablets, glass vials Protease Inhibitor Cocktail and cOmplete™ His-Tag Purification Resin were from Roche (Germany). Acetic acid ≥99.0% (T) and skimmed milk powder were obtained from Fluka Analytical (Switzerland). Triton^®^ X-100, sodium carbonate and ammonium persulfate were obtained from Sigma-Aldrich (Germany). HEPES and Brilliant Blue R were obtained from Sigma (UK). Sodium dodecyl sulphate was obtained from BDH Laboratory (UK). 30% Acrylamide/Bis Solution, 37.5:1, Precision Plus Protein™ Dual Xtra Standards, Precision Protein StrepTactin-HRP Conjugate and Clarity Western enhanced chemiluminescence (ECL) Substrates were obtained from Bio-Rad Laboratories (USA). Mouse Anti-Hepatitis B Virus Antibody, core antigen (ayw), clone 10E11 was from Merck Millipore (USA). HCL was obtained from Aeros Organic (Germany). Anti 6-His affinity purified antibody was obtained from Bethyl Laboratories Inc. (USA). SnakeSkin™ Dialysis Tubing, 10 K MWCO was from Thermo Scientific (USA). *Escherichia Coli*, BL21 (DE3) was obtained from Dr. Mitla Garcia Maya, Randall Division of Cell and Molecular Biophysics, King’s College London, UK. Plasmid pET-22b(+)-WT-HBc-His6 encoding C-terminally His tagged HBc protein (residues 1–183) Accession No. CAA59538 was obtained from Associate Professor Chiaki Ogino, Chemical and Science Engineering Department, Kobe University, Japan.

## Methods

### Expression, purification and assembly of WT-HBc particles

For gene expression of WT-HBc protein, the plasmid for expression of WT-HBc (pET-22b(+)-WT-HBc-His6); was transfected into *E.coli* BL21 (DE3) strain. *E. coli* transfected WT-HBc was expressed, purified and assembled *via* the following methods:

#### Conventional method

*E. coli* transfected WT-HBc (15 μl) was cultured in 10 ml of LB media in the presence of 100 μg/ml ampicillin and grown at 37 °C for 16 h using an incubator shaker. The culture was then diluted with 500 ml of fresh LB media in the presence of 100 μg/ml ampicillin and grown to OD_600_ 0.7–0.8 at 37 °C. The culture was induced by adding isopropyl-β-thiogalactopyranoside (IPTG) to a final concentration of 0.1 mM at 25 °C overnight. Cells were harvested at 4500 g, 4 °C for 15 min. Pelleted cells were re-suspended in the 30 ml of lysis buffer (50 mM Tris, 100 mM NaCl, 5 mM EDTA, 0.2% v/v Triton X-100, pH 8.0). The cells were lysed on ice by three cycles of probe sonication for 1 min each with 1 min intervals to avoid heating the material. The supernatant was removed by centrifugation at 18,500 g, 4 °C for 30 min. The core particles in the cell pellet were washed in 30 ml of lysis buffer and collected by centrifugation at 18,500 g, 4 °C for 30 min. The cell pellet containing WT-HBc particles was denatured in 40 ml of dissociation buffer (4 M urea, 200 mM NaCl, 50 mM sodium carbonate, 10 mM 2-mercaptoethanol, pH 9.5) by overnight incubation at 4 °C. Then, the pellet was discarded by centrifugation at 18,500 g, 4 °C for 30 min.

Soluble fraction containing contaminating proteins was separated from HBc particle proteins using Ni^2+^-chelate affinity chromatography. A column with 6 ml of cOmplete™ His-Tag Purification Resin was equilibrated with 3-times bed-volume (18 ml) of dissociation buffer. The column was loaded with the protein probe and washed with 18 ml of dissociation buffer. Bound HBc particle proteins were eluted with 14 ml of elution buffer (4 M urea, 200 mM NaCl, 50 mM sodium carbonate, 10 mM 2-mercaptoethanol, 1 M imidazole, pH 9.5). The eluted material was collected in 1 ml fractions. The aliquots of each fraction were subjected to sodium dodecyl sulphate-polyacrylamide gel electrophoresis (SDS-PAGE) and stained with Coomassie Brilliant Blue (CBB) to analyse their purity.

Fractions containing the HBc protein were re-assembled to particles by the removal of the urea in assembly buffer (500 mM NaCl, 50 mM Tris, 0.5 mM EDTA, pH 7.0). Specifically, protein fractions were dialysed against 5 l of dialysis buffer using SnakeSkin™ Dialysis Tubing, 10 K MWCO at 4 °C for overnight. Assembled HBc particles were filtered using 0.44 μm filter to remove any aggregates.

#### Improved method

*E. coli* transfected WT-HBc (15 μl) was cultured in 10 ml of AIM-TB media (10 g/l tryptone, 5 g/l yeast extract, 3.3 g/l (NH_4_)_2_SO_4_, 6.8 g/l KH_2_PO_4_, 7.1 g/l Na_2_HPO_4_, 0.5 g/l glucose, 2.0 g/l α-lactose, 0.15 g/l MgSO_4_) in the presence of 100 μg/ml ampicillin and grown at 37 °C for 16 h using an incubator shaker. The culture was then diluted with 500 ml of fresh AIM-TB media in the presence of 100 μg/ml ampicillin and grown at 25 °C for 72 h. Cells were harvested at 4500 g, 4 °C for 15 min. Pelleted cells were re-suspended in the 30 ml of lysis buffer (50 mM Tris, 100 mM NaCl, 5 mM EDTA, 0.2% v/v Triton X-100, 1x cOmplete™ protease inhibitor pH 8.0). The cells were treated with RNase A at a final concentration of 5 μg/ml at 4 °C overnight. The lysate was sonicated using a probe sonicator on ice by three cycles for 1 min each with 1 min intervals to avoid heating the material. The supernatant was removed by centrifugation at 18,500 g, 4 °C for 30 min. The core particles in the cell pellet were washed in 30 ml of lysis buffer and collected by centrifugation at 18,500 g, 4 °C for 30 min. The cell pellet containing WT-HBc particles was denatured in 40 ml of dissociation buffer (8 M urea, 200 mM NaCl, 50 mM sodium carbonate, 10 mM 2-mercaptoethanol, pH 9.5) by overnight incubation at 4 °C. Then, the pellet was discarded by centrifugation at 18,500 g, 4 °C for 30 min.

The soluble fraction containing-contaminating proteins were separated from HBc particle proteins using Ni^2+^-chelate affinity chromatography. A column with 6 ml of cOmplete™ His-Tag Purification Resin was equilibrated with 3-times bed-volume (18 ml) of dissociation buffer. The column was loaded with the protein probe and washed with 18 ml of dissociation buffer. Bound HBc particle proteins were eluted with 14 ml of elution buffer (2 M urea, 200 mM NaCl, 50 mM sodium carbonate, 10 mM 2-mercaptoethanol, 1 M imidazole, pH 9.5). The eluted material was collected in 1 ml fractions. The aliquots of each fraction were subjected to SDS-PAGE and stained with CBB to analyse their purity.

Fractions containing the HBc protein were re-assembled to particles by the removal of urea. Specifically, protein fractions were dialysed against 2 l of dialysis buffer 1 (0.5 M urea, 100 mM Tris, 150 mM NaCl, 2 mM DTT, 1 mM EDTA, 10 mM CaCl_2_, pH 8.0) using SnakeSkin™ Dialysis Tubing, 10 K MWCO at 4 °C for 4 h, allowing HBc protein to start assembled. Then, the solution was dialysed against dialysis buffer 2 (100 mM Tris, 150 mM NaCl, 1 mM EDTA, 10 mM CaCl_2_, pH 8.0) to completely removed urea and DTT, hence assembled HBc particles. HBc particles were filtered using 0.44 μm filter to remove any aggregates.

All protein concentrations were measured using NanoDrop™ ND-1000 UV-Vis Spectrophotometer.

### Tapping mode Atomic Force Microscopy (TM-AFM) analysis of purified WT-HBc particles

A 10 μg/ml sample of purified WT-HBc particles (100 μl) was deposited on mica surfaces for 5 min and then flushed with air. TM-AFM on the mica substrates were carried out in air at 25 °C using a Bruker Dimension ICON with Scan Assist. The surfaces were imaged with a general purpose-tapping tip made by MikroMasch in Estonia (NSC15/no Al, tip radius <10 nm; tip height = 20–25 μm; cone angle <40°; cantilever thickness = 3.5–4.5 μm; cantilever width = 32–28 μm; cantilever length = 120–130 μm; frequency *f*_0_ = 265–400 kHz; force constant k = 20–75 N m^−1^, VEECO, USA). The statistical analysis of the AFM images was carried out using WSxM v5.0 Developed 6.2 software (Spain).

### SDS-PAGE and western blot analysis of WT-HBc particles

The expression of each WT-HBc monomer was confirmed by western blotting. The purified WT-HBc particles were analysed by SDS-PAGE and electro-transferred onto a nitrocellulose membrane. For the detection of the His6-tag, rabbit anti-6-His antibody was used as a primary antibody at 1:1000 dilution followed by HRP-linked anti-rabbit at 1:1000 dilution. For the detection of the HBc, mouse anti-HBc antibody was used as a primary antibody at 1:1000 dilution, followed by HRP-linked anti-mouse at 1:1000 dilution. For staining of the ladder, Precision Protein StrepTactin-HRP Conjugate was used at 1:10,000 dilution. The specific bands were detected with enhanced chemiluminescence (ECL) detection system. The membrane was imaged using the ChemiDoc^TM^MP and analysed with Image Lab 4.1 software.

### Circular Dichroism (CD) for protein secondary structure analysis

CD thermal scan measurements were performed on a Chirascan spectrometer (Applied Photophysics, Leatherhead, UK) supplied with a thermoelectric temperature control system. Temperature-dependent conformational changes were measured for WT-HBc particles (0.1 mg/ml) in Tris buffer (5 mM, pH 8.0). CD spectra of the samples were recorded from 260 to 180 nm using a 0.5 mm cuvette at 6 °C before starting the thermal scan. The temperature sensitivity was then tested by increasing the temperature from 6 to 94 °C at 1 °C/min heating rate and 2 °C/step. At the end of the thermal scan, the sample was equilibrated at 94 °C and the CD spectrum was recorded. Once the thermal scan was completed, the samples were cooled to 6 °C and equilibrated for 15 min before recording the CD spectra. Data analysis was performed using Applied Photophysics Chirascan software, and the transition temperatures were determined with Global 3 analysis software for dynamic multimode spectroscopy.

### Statistics

For all experiments, data were presented as mean ± SD, where n denotes the number of repeats. Independent variable Student t tests were performed using one-way ANOVA. The t-value, degrees of freedom and two-tailed significance (p-value) were determined. *p < 0.05, **p < 0.01 and ***p < 0.001.

## Additional Information

**How to cite this article**: Bin Mohamed Suffian, I. F. *et al*. Yield Optimisation of Hepatitis B Virus Core Particles in *E. coli* Expression System for Drug Delivery Applications. *Sci. Rep.* **7**, 43160; doi: 10.1038/srep43160 (2017).

**Publisher's note:** Springer Nature remains neutral with regard to jurisdictional claims in published maps and institutional affiliations.

## Supplementary Material

Supplementary Information

## Figures and Tables

**Table 1:** Yield and nucleic acid contamination characterisation of HBc particles prepared using “Conventional” or “Improved” methods.

Method	HBc code	Protein yield (mg)^[1],[2]^	OD_260_:OD_280_^[1],[2]^
Conventional	WT-HBc (C)	1.21 ± 0.3	1.02 ± 0.04
Improved	WT-HBc (I)	3.21 ± 0.21***	0.58 ± 0.01***

^[1]^ Values were obtained with Nanodrop from 1 litre bacteria containing media normalised to LB media.

^[2]^ Results are expressed an average ± SD (n = 3). ^***^p = 0.00024 (<0.001) (One-way ANOVA).
