# Supplementary material for: Yield Optimisation of Hepatitis B Virus Core Particles in E. coli Expression System for Drug Delivery Applications
Source: Sci Rep. 2017 Mar 3;7:43160. doi: 10.1038/srep43160 (PMC5335696; doi:10.1038/srep43160)
Supplement: Supplementary Information [file srep43160-s1.pdf]

Supplementary Information

Yield Optimisation of Hepatitis B Virus Core Particles in *E. coli* Expression System for Drug Delivery Applications

Izzat Fahimuddin Bin Mohamed Suffian[1], Mitla Garcia-Maya[2], Paul Brown[2], Tam Bui[3], Yuya Nishimura[4], Amir Rafiq Bin Mohammad Johari Palermo[5], Chiaki Ogino[4], Akihiko Kondo[4], Khuloud T. Al-Jamal*[1]

[1] Institute of Pharmaceutical Science, King's College London, Franklin-Wilkins Building, 150 Stamford Street, London SE1 9NH, UK.

[2] Randall Division of Cell & Molecular Biophysics, King's College London, New Hunt's House, London SE1 1UL, UK.

[3] Biomolecular Spectroscopy Centre, King's College London, The Wolfson Wing, Hodgkin Building, SE1 1UL, UK.

[4] Department of Chemical Science and Engineering, Graduate School of Engineering, Kobe University, 1-1 Rokkodai, Nada, Kobe 657-8501, Japan.

[5] Barts and The London School of Medicine and Dentistry, Queen Mary University of London, 4 Newark Street, London E1 2AT, UK.

Correspondence to:
Professor Khuloud T. Al-Jamal
Institute of Pharmaceutical Science, King's College London, Franklin-Wilkins Building,
150 Stamford Street, London SE1 9NH, UK.
Tel: +44(0)20-7848-4525

Supplementary Information

1. Supplementary Figures

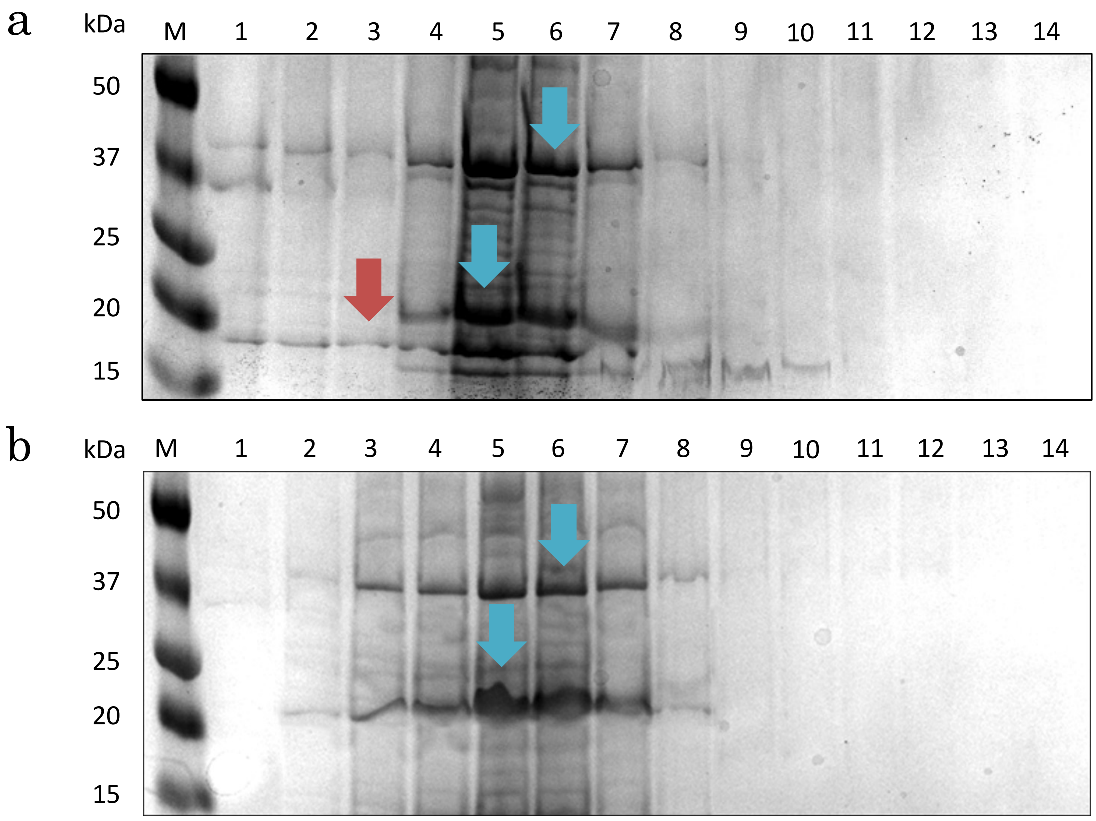

Figure S1. Elution profile of WT-HBc monomers from Ni^{2+}-chelate affinity chromatography column. A column with 6 ml of cOmplete™ His-Tag Purification Resin was equilibrated with 3-times bed-volume (18 ml) of the dissociation buffer. The column was loaded with the protein probe and washed with 18 ml dissociation buffer. Bound proteins were eluted with 14 ml of elution buffer and collected in 1 ml fractions. The aliquots of each fractions were subjected to SDS-PAGE gel electrophoresis and stained with Coomassie Brilliant Blue. M, marker in kDa; Numbers 1-14 represent aliquots of the respective elution fractions for **(a)** WT-HBc (C) or **(b)** WT-HBc (I) monomers. Blue arrows represent WT-HBc monomers (21 kDa) and dimers (42 kDa). Some unknown protein bands were observed at molecular weight lower than 20 kDa (red arrow).

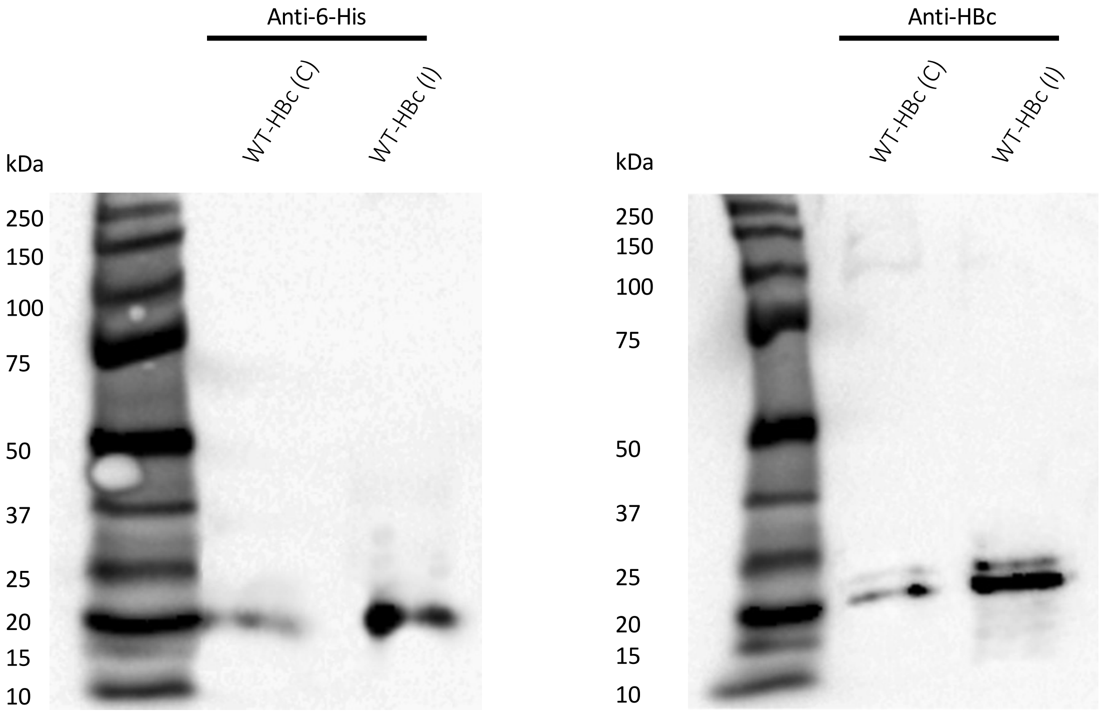

Figure S2. Protein specificity of HBc particles (full length blots). Western blotting of WT-HBc particles**.** Denatured HBc samples were subjected to SDS-PAGE followed by immuno-blotting using anti-6-His and anti-HBc antibodies. Results confirmed the presence of specific protein bands at 21 kDa. CD graph shows the overall conformation of far-UV CD analysis of WT-HBc core particles.
